# *'Pregnancy comes accidentally - like it did with me': *reproductive decisions among women on ART and their partners in rural Uganda

**DOI:** 10.1186/1471-2458-11-530

**Published:** 2011-07-05

**Authors:** Rachel King, Kenneth Khana, Sylvia Nakayiwa, David Katuntu, Jaco Homsy, Pille Lindkvist, Eva Johansson, Rebecca Bunnell

**Affiliations:** 1 Global Health Sciences, University of California, San Francisco, 50 Beale St, San Francisco, CA 94105, USA; 2 Department of Public Health Sciences, Karolinska Institute, Stockholm, Sweden; 3 Division of Global HIV/AIDS, Center for Global Health, Centers for Disease Control and Prevention (CDC), Entebbe, Uganda

**Keywords:** HIV, ART, Uganda, HIV-infected persons, Africa, reproductive intentions

## Abstract

**Background:**

As highly active antiretroviral therapy (ART) restores health, fertility and sexual activity among HIV-infected adults, understanding how ART influences reproductive desires and decisions could inform interventions to reduce sexual and vertical HIV transmission risk.

**Methods:**

We performed a qualitative sub-study among a Ugandan cohort of 1,000 adults on ART with four purposively selected categories of participants: pregnant, not pregnant, delivered, and aborted. In-depth interviews examined relationships between HIV, ART and pregnancy, desire for children, perceived risks and benefits of pregnancy, decision-making regarding reproduction and family planning (FP) among 29 women and 16 male partners. Analysis focused on dominant explanations for emerging themes across and within participant groups.

**Results:**

Among those who had conceived, most couples stated that their pregnancy was unintentional, and often occurred because they believed that they were infertile due to HIV. Perceived reasons for women not getting pregnant included: ill health (included HIV infection and ART), having enough children, financial constraints, fear of mother-to-child HIV transmission or transmission to partner, death of a child, and health education. Most women reported FP experiences with condoms and hormonal injections only. Men had limited FP information apart from condoms.

**Conclusions:**

Counselling at ART initiation may not be sufficient to enable women who do not desire children to adopt relevant family planning practices. On-going reproductive health education and FP services, with emphasis on the restoration of fertility after ART initiation, should be integrated into ART programs for men and women.

## Background

Major strides have been made in recent years in expanding access to antiretroviral therapy (ART) and comprehensive care for HIV-infected men and women in sub-Saharan Africa. For many people living with HIV, ART enables a return to normal life including a resumption of sexual activity and a new or renewed desire for children. This desire is often fueled by the strong societal and traditional values attached to parenthood in sub-Saharan Africa and is further enhanced by the development of increasingly effective antiretroviral regimens to reduce the risk of HIV transmission from an infected mother to her newborn or breastfeeding child [1-6]. However, these regimens and the associated care represent only two of the four pillars of the WHO global effort to prevent mother-to-child HIV transmission (MTCT). The two other pillars include the primary prevention of HIV infection in women of child-bearing age, and the prevention of unwanted pregnancies in HIV-infected women [1].

A complex web of biomedical, cultural, and socio-economic factors influences fertility desires, reproductive decision-making, and incident pregnancy among HIV-infected women and men [5,6]. Globally, studies have documented different roles that knowledge of HIV diagnosis plays in decision-making concerning conception as well as pregnancy resolution [3,7-13]. Factors associated with desire for pregnancy among HIV-infected women have included cultural and societal expectations around motherhood, MTCT and ART programs, while fears of partner and infant infection, having previously had an infected baby, and community disapproval have been associated with men's and women's wishes to avoid pregnancy [3]. Kisakye et al, exploring factors underlying pregnancy decisions among HIV-infected pregnant women delivering at the national referral hospital in Kampala, Uganda, found that awareness of MTCT risk reduction strategies, disclosure of HIV status and awareness of spouse's HIV sero-status, availability of ART, stigma about HIV and childlessness, influence of partners and family members, and the health status of the mother and her family members were all factors that motivated HIV-positive women to conceive or influence pregnancy decisions in spite of their sero-status [2].

Initial studies among women on ART showed low desire for children and high incidence of pregnancy over up to 2 years of follow-up in Uganda [14,15]. Additional reports by Myer and colleagues summarizing data from 4,500 women in eleven PMTCT programs in seven sub-Saharan African countries over 4 years of follow-up after ART initiation confirmed that ART use was associated with increased incidence of pregnancy[16]. In South Africa, fertility intention among men and women on ART has been positively associated with male gender, having fewer children, being in a relationship of less than 5 years, and increasing duration of ART for women but not for men [7]. Kaida et al. have proposed a conceptual framework that describes these biological, behavioral, economic and social factors that could influence ART's effects on fertility [17].

Few qualitative studies have explored the factors associated with pregnancy among women on ART and their partners. We previously reported low desire for children and high rates of unintended pregnancy in a prospective cohort study of 718 HIV-infected women followed-up for 24 months after ART initiation in rural Uganda [14]. To help understand in greater depth the quantitative results and to inform the development of more effective and comprehensive approaches to integrated reproductive health and HIV care, we conducted a qualitative study among these women and some of their partners and examined the influences that shaped the reproductive decisions of these individuals, the choices they had, and how these decisions were played out.

## Methods

We conducted a qualitative sub-study with participants enrolled in the Home-Based AIDS Care (HBAC) project in rural Eastern Uganda between September 2006 and June 2007. HBAC delivered free HIV and TB care and support services to the homes of approximately 1,000 participants in the project catchment area that covered a 100 km radius around Tororo town. Subsistence agriculture was the main livelihood of the people in the area; nearly half of them lived below the poverty line and the majority had not received education beyond primary school level (Uganda Bureau of Statistics 2003).

HBAC participants were recruited in 2003 from The AIDS Support Organisation (TASO), a non-governmental organization that has provided care and support services for HIV-infected people in Uganda and the Tororo area since 1987. TASO clients >18 years-old and with a CD4 cell count <250 cells/μL or in WHO disease stage 3 or 4 were offered first-line ART. HBAC study participants were enrolled at the HBAC study clinic between May 2003 and June 2006, and received ART and tuberculosis drugs at their homes on a weekly basis. No future routine clinic visits were scheduled but participants could come or be referred to the clinic or hospital for treatment of symptoms. Research counselors visited participants quarterly to collect behavioral data and to provide ongoing support on adherence to ART and sexual risk behavior. They also provided individual or couple home-based HIV counseling and testing to all household members of HBAC participants, including spouses. Confidentiality was emphasized in all counseling sessions.

Prior to HBAC, advice and counseling had been given by TASO on condom use as well as on abstinence as the most effective HIV transmission prevention strategies for sexually active persons. On joining HBAC, participants were provided again with information detailing a range of HIV risk reduction options including condom use, abstinence, partner testing, disclosure of HIV status to current or new/potential sexual partners, being faithful to one HIV-tested partner, reducing the number of sexual partners, reduced frequency of sex, alternative forms of sexual expression, and treatment of STIs. In addition, study participants were counseled at enrolment about the potential effects of ART on restoring health, fertility and sexual activity, and were referred to the hospital family planning (FP) clinic adjacent to the study clinic if interested in using FP other than condoms. The FP clinic provided standard FP counseling and FP services for as many clinic visits as necessary. Contraceptives were supplied by the Ministry of Health and included combined oral and progesterone-only pills, hormonal injectables and implants, intra-uterine devices and tubal ligation and condoms. Condoms were also available for free from the FP clinic as well as from all HBAC clinicians, counselors and home visitors, both at the study clinic and during home visits.

In-depth interviews were conducted among 29 HBAC women on ART and 16 of their partners to explore personal beliefs and experiences. Participants were purposefully selected to provide a range of views and were based on ART and pregnancy status in the last 12 months. In all they included: 21 women on ART who had become pregnant and/or had delivered or aborted in the last 12 months and 11 of their partners; and 8 women who had not become pregnant and 5 of their partners. In the quotes reported in the 'results section', participants (women and men) were labeled as either 'pregnant', 'not pregnant', 'aborted', or 'delivered'. Counselors attempted to locate all partners of women selected for the study, but not all women had partners able to participate. The 16 male partners interviewed were those who not only agreed to participate; but also were in an ongoing and/or stable relationship with their partners; and were available for the interviews. No women refused to be interviewed. Data responses for key themes reached saturation with the 45 selected respondents.

Interviews were carried out in the participants' native languages and lasted about 1 1/2 hours. Open-ended questions included: number of living children at home, desire for children and factors related to pregnancy, reactions to pregnancy, relationship with partner, experience with death of a child, relationship between ART and pregnancy, and experiences with family planning. In-depth interviews were transcribed, translated into English, and coded by an analysis team. Guidelines that included three distinct analysis stages were used for thematic coding as the primary analytic strategy [18]. After reading two transcripts, the analysis team members collaboratively developed a codebook of themes based on the interview topics as well as those emerging from the data. Two more transcripts were then reviewed to include additional topic areas and themes. This process of thematic coding was repeated five times until the codebook reached a stage where no new themes or topic areas emerged [18]. To ensure inter-rater consistency, the analysis team compared their individual coding of the same transcripts and a coding concordance was calculated. All transcripts were then coded using the final version of the codebook and merged using NVivo software (version 2.0, QSR International Pty. Ltd, Victoria, Australia). After coding, the merged project was transferred to NVivo version 7 for further management. Themes were summarized across participants, and analysis focused on identifying dominant explanations. Interactive discussions were held with the analysis team, who were from diverse backgrounds (physician, social scientists, counselors) to validate data interpretations and resolve any discrepancies.

The study was approved by the Institutional Review Boards of the Uganda Virus Research Institute, Uganda, and the Centers for Disease Control and Prevention, Atlanta, Georgia, USA. All clients provided written informed consent for interview and recording.

## Results

### Sample characteristics

We interviewed 29 women and 16 of their sexual partners. Participant characteristics are shown in Table 1: 69% (20/29) of the women and 75% (12/16) of the men were married or co-habiting. Their mean ages were 37 and 32 years old respectively. Eight women were pregnant at the time of the interview, seven had delivered within the past year, six had aborted and eight had not become pregnant since starting ART. Of the 16 men, 3 were partners of pregnant women, 5 of women who had not become pregnant since on ART, 2 were partners of women who had aborted and 6 of women who had delivered an infant in last year.

**Table 1:** Characteristics of Study Participants; HBAC Qualitative Pregnancy Study; EasternUganda; 2006-2007 (N = 45)

	Women N = 29 (%)	Men N = 16 (%)	All N = 45
**Mean Age**	32 yrs	37 yrs	33.5 yrs

**Marital Status**			

• Married/co-habiting	20 (69)	12 (75)	32 (71)
• Single/separated	3 (10)	1 (5)	4 (9)
• Widowed	6 (21)	3 (20)	9 (20)

**Pregnancy Status**			

• Pregnant	8 (23)	3 (20)	10 (22)
• Not pregnant	8 (28)	5 (31)	13 (29)
• Aborted	6 (21)	2 (11)	8 (18)
• Delivered	7 (28)	6 (38)	14 (31)

**Number of living biological children**			

• 0-2 children	11 (38)	5 (31)	16 (36)
• 3-4 children	11 (38)	5 (31)	16 (36)
• >4 children	7 (24)	6 (38)	13 (28)

**Number of parents who have children who have died**			

• 0-2 children died	24	12	36
• 3-4 children died	5	4	9

### Reasons for pregnancy

**Figure 1:**
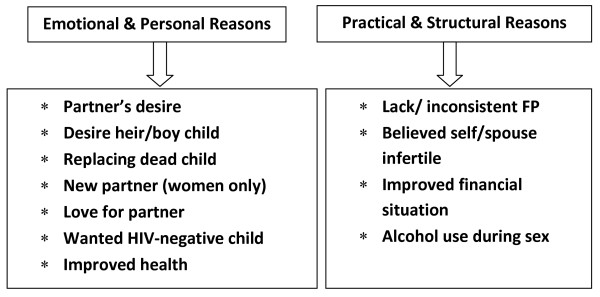
**Themes and sub-Themes Associated with Becoming Pregnant among Women and Men in HBAC Qualitative Pregnancy Study; Eastern Uganda, 2006-2007**.

Most of the individuals (women and partners) from the categories of pregnant, aborted or had delivered recently, stated clearly that the pregnancy was unintended. Many mentioned that the current pregnancy was an accident and a surprise because they believed that they were infertile following long periods of amenorrhea or the absence of pregnancy despite having unprotected sex. However, individuals also mentioned multiple motivations for pregnancy, with the main reasons often intertwined in a number of interactions between human relations, health, social, economic and cultural factors. Two broad categories emerged from this tangled web of motivations: emotional/personal and practical/structural reasons (Figure 1). The motivations in each category were often mentioned by both men and women though at times the way in which the idea was expressed was different for men or women. Some issues were expressed more frequently by either men or women and there were, in addition, some gender-specific explanations.

### Emotional and personal reasons for pregnancy

Many more women than men cited their partner's desire for children as an important factor associated with pregnancy. Partner desire was often interconnected with cultural expectations on child-bearing, alcohol use, desire for sex without a condom or desire for a male heir. In some couples, partner desire for children was confirmed when the man was interviewed. In other cases, only one member of the couple claimed partner desire for a child. One man also stated that having a wife implies automatic child bearing.

*Now I think she [*wife*] must have wanted a child, unbeknown to me[*...*], it seems that she wanted. But I didn't want [a child]. Right from the time we started bearing children, it occurred accidentally. You just discover that the person has already conceived. It wasn't that I intended to go on giving birth to children up to that number. When you get a wife, know that any time you must give birth to children*... *I felt that once we bear about four children, we would stop *(man with 6 living children, 0 deceased, wife delivered).

This man also described how in his home, discussion did not occur about whether or not to have another child; rather, pregnancy just happened.

#### Desire for male child or heir

Men mentioned that they wanted to have one child that is HIV-negative or a male as only boys can inherit the family land and the family name in this cultural context. This reason was sometimes combined with wanting to replace children who had died.

*It is believed that when one dies without a child, then that life is gone wasted. And even biblically when you die without a child it means that your generation stops/ends there *(man with one living girl child; 4 deceased; wife delivered).

The same couple reasoned that since their health had improved they could now try for an heir.

*We looked at the number of children we had (*one*) since we lost some children, this changed my desire to have children even after knowing our [HIV] status *(man with one living child; 4 deceased; wife delivered).

#### New partner

Another personal reason for pregnancy included acquisition of a new partner which culturally entails a new child. One woman clearly articulated how she was counseled about family planning at ART initiation yet at that time she was sure she would never get pregnant. However, as her health improved, her sexual desire returned and she initiated a new relationship. The new partner began helping her financially and she became pregnant.

*[...] the new partner used to help me and provide me what I wanted and that is how it all happened *(woman with 2 living children, 4 deceased, aborted).

#### Religion

More men than women mentioned that it was God that made decisions regarding children;

*[...] then all of a sudden someone is pregnant. It is God who takes the upper hand not any of us *(man with 3 living children, 0 deceased).

One man and one woman mentioned that love for their partners was the reason for pregnancy.

### Practical and structural reasons for pregnancy

#### Belief in being infertile/lack of family planning

Many pregnancies were reported as a surprise because women and/or their partners believed that they were infertile following long periods of amenorrhea or the absence of pregnancy despite having unprotected sex after their HIV diagnosis. Both men and women mentioned a lack of or inconsistent use of family planning as a logical consequence of their belief that they or their partners were infertile.

*[...] I thought my first wife couldn't get pregnant again after staying for six years without having a baby and for the second one, we were relying on counting days when she was to be on her M.P. [*menstrual period*] and use of condoms once in a while. That's why pregnancies took us by surprise *(man with 3 living children, 2 deceased; wife delivered).

Other women reported disappointment at becoming pregnant after discontinuation of family planning.

*Initially I had tried to go for injectable (Depo) to some clinic where they charged me. But I could not continue because we lacked money and during this period we had no condoms [...] I realized I was pregnant *(woman with 3 living children; 1 deceased, pregnant).

#### Financial situation

Some women mentioned that financial dependence on their partner was their reason for becoming pregnant. Gender/power issues were entwined with social and financial status and revealed how women expressed their ability to negotiate condom use with new partners in particular.

*[...] this man promised to help me and my children. But as it is, a man cannot simply give you all the help when [...] you do not have his kid *(widowed woman with 5 living children, none deceased; pregnant).

*I tried to suggest to my [new] sexual partner to use condoms [...] he refused and I accepted to have unprotected sex because he was very supportive [*financially*]. He would bring domestic necessities and there was no way I could disobey him *(widowed woman with 3 living children, none deceased; delivered).

One man discussed his conflicting emotions about wanting an uninfected heir on the one hand and questioning on the other hand his and/or his wife's ability to raise such a child.

*I really wanted her to get pregnant because I felt I wanted to try and have a child who would be HIV-negative. I felt the pressure within myself that I should have a child so that when I die he could take over my estates. You first look at your state especially us on ARVs, are you able to look after the child after breastfeeding. One needs to look at the economic base before you make the decision *(man with 1 living child, 4 deceased, wife delivered).

#### Alcohol

Five women and one man explained the effect of alcohol and disinhibition when drunk. One woman recounted her situation in relation to gender/power relations.

*My husband came home drunk and demanded sex without a condom. He reasoned that we were both infected so having sex with condoms was not protecting us from anything. I tried to insist [*on condoms*] but was overpowered being a woman in the man's house *(woman with 4 living children; 4 deceased, delivered).

### Reasons For Not Getting Pregnant

**Figure 2:**
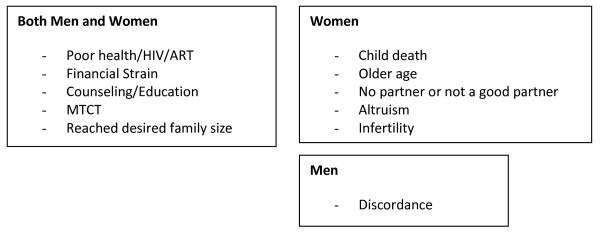
**Themes Associated with not Becoming Pregnant among Women and Men in HBAC Qualitative Pregnancy Study; Eastern Uganda, 2006-2007**.

To compare responses, women and their partners who did not get pregnant since initiation of ART were also interviewed. Among those who did not get pregnant, HIV infection/ill health/and taking ARVs were the main motivations mentioned by women and their partners (Figure 2).

*I knew if I was to have sex, then the condom has to be used because I do not want re-infection from him and I cannot afford getting pregnant to reduce my life span *(woman with 3 living and 2 deceased children, not pregnant).

*It is because when they first tested my CD4 they said I was weak. If I am to give birth, I don't know what I will give birth to [*will it live*?]. Besides, whom am I giving birth for? I want my children to reach a certain level. If I am to think about the last child I had, I did not know I would live to date? It's a miracle. That is why I do not want to have another child or even talk about it *(woman with 4 living children and 1 deceased, not pregnant).

Having reached their desired family size, financial constraints, fear of mother-to-child HIV transmission or of HIV transmission to partner, death of a child, lack of partner and educational messages received from counselors or doctors were additional reasons stated for not getting pregnant (Figure 2).

*Given the fact I had lost all my children, I didn't want to grieve again *(woman who lost 4 children, not pregnant).

*I kept thinking about the miscarriage I had before; the pain I went through. I wondered how I would be this time. Life became so miserable [...] I felt like committing an abortion or even dying. I kept thinking that even if the child lived, it would live a desperate life because it will also suffer like I have suffered *(woman with 1 living child; 3 deceased; aborted).

Some women expressed clear decision-making regarding their next pregnancy, often based on their resources and relationship with partner.

*Right now, pregnancy is not intended. It requires when you have got your man who is responsible and dependable *(widowed woman with 3 living children and 3 deceased, not pregnant).

Men had similar reasons related to financial status. One mentioned, "*we thank god for having carried her through although it is very problematic to feed them [*the children*]" *(man with 2 living children, 0 deceased, wife delivered).

#### Influence of ART on reproductive decision making

Men and women gave positive and negative responses regarding the influence of ART on their reproductive-decision making. Most of them noted that improved physical and emotional health due to ART had resulted in increased desire for sex and thus pregnancy in some cases; a few mentioned as well that becoming stronger and healthier allowed them to look after children much better and to plan for the future.

*The drugs make us regain our normal strength that we tend to forget about the fact that we are infected. As for me, I do not even remember that I am infected? Life is so normal I go on my daily work like I used to do *(woman with 3 living children, 2 deceased, aborted).

*There is a category [*of women*] that has lost all their children. Such women when ARVs help them look better,they feel cheated not to produce. So when they do not have one, then everything loses meaning *(man with 3 living children, 0 deceased, wife not pregnant).

Some men and women, noted that ARVs have not had any effect on their sexual desire or desire for children. One of the reasons provided for no effect was that improved health cannot override a firm decision to have no more children. A man who was a partner of one of the women who had not gotten pregnant mentioned that;

### Family Planning

As shown in Table 2, women and men described experiences with condoms and injectable medroxyprogesterone acetate (MPA or DepoProvera) and expressed interest in using more permanent contraceptive methods (specifically tubal ligation). Reasons for discontinuation of injectable hormonal methods were mainly cost and side effects such as heavy bleeding. For condoms, reasons for discontinuation generally centered around aspects of negotiation with male partner. Women and men cited benefits of both MPA and condoms including the fact that women can use MPA without their partners' awareness. Many participants, both men and women, mentioned that they, or their partner, wanted to get a tubal ligation because of its permanence.

**Table 2:** Family Planning Experiences and Intentions of Women and their Partners in HBAC Qualitative Pregnancy Study; Eastern Uganda, 2006-2007

	PAST	CURRENT	FUTURE
	Reasons for changing methods	Experiences with method	Reasons for decision
**Woman**	Injections: side effects *They were giving me problems. I was bleeding till the end of the month *(woman who is not pregnant with 0 childre*n)*	Injections: good *...using for ten months now, and I feel okay and it works ....because they told me it has no side effect. I do it without letting him know. He only learns later (woman with *2 living children, not pregnant)	Injections: fear of pain with TL *My friends say when they were operating her she felt pain and so I do not want to go in for it. Am considering the use of injections as I think I cannot forget and it cannot strain me at all*.(woman with no children)
	Injections: cost *I went when to some clinic in Mukujju centre and they told me to pay money. Well I had to pay but it became a challenge as time went on in that I could not get the money at the exact day I was meant to go. And so I missed and then left it at that. *(pregnant woman with 3 children)		

	Condoms*: *male partner rejection *I tried the condom but the man does not want it that is why I have now opted for Tubal-ligation. (aborted with 3 children)*	Condoms: good *Everything is fine. We have never realized any thing bad or disturbing. Actually we got used to them to extent that it has become the normal way of having sex*.	Condoms *It is just the condoms and nothing else. I am proud to have succeeded in my strategy of using the condoms every time we have sex. I actually ensure that he has the condom on before we proceed *(woman with 3 living children)
	*What I wanted was to stop because my husband at times you can agree with him to use condoms, then at a later stage he refuses and he starts beating me all the time *(pregnant woman, 2 living children*)*	*I feel proud because I can live healthy, not infecting my husband yet very sure of not increasing the number of children we have*. (3 children + 3 non-biol children, not pregnan*t)*	
	
			Tubal ligation: permanence *It is good because you do not get pregnancies like this one. The best is TL because there is no way you can get back to a situation as bad as mine. I am looking forward to delivering and having the TL done. I do not have time to listen to any other method. *(pregnant; 3 living children)

**Man**	Injection: side effects *My wife told me that she used an injection and it interfered with her cycle of menstruation. At times she would get this flow for a long time. I personally did not like this*. (man whose wife delivered; 4 living children, 2 deceased)	Injection: good *She is using Depo injection and she has used it for almost one year now. Before she started using it, she was fat and now as she is using it, she has started losing weight. *(man with 1 living child, 4 deceased, delivered*)*	
	Condom; lack of access *we were using but they got finished and this later created this pregnancy *(man whose wife aborted; 6 living children)	Condom *We agreed on using condoms whenever we have sex until we die. I had even never seen a condom before. But because the counselor told us the necessity of condoms, it became something admirable *(man whose wife is not pregnant; 3 children)	Condom-limitations of injections *I was told that also the injection may expire at a certain time and we discover that she is pregnant. That is why the doctors now advised that we be using condoms*. (man whose wife delivered; 6 living children)
			Tubal ligation: permanence *the burden of looking after the children, re-infecting ourselves and not wanting to risk having another child who is positive*. (man whose wife is pregnant; 3 living children, 2 deceased*)*
			*We chose tubal ligation because it is permanent, there is no need to get to another one that needs to revisit it after a time. *(man whose wife is pregnant; 6 living children, 2 deceased)

*It [*tubal ligation or TL*]is good because you do not get pregnancies like this one. The best is TL because there is no way you can get back to a situation as bad as mine. I am looking forward to delivering and having the TL done. I do not have time to listen to any other method *(woman with 3 living children, 2 deceased, pregnant).

## Discussion

This qualitative study of 29 women on ART and 16 of their partner show that most women who became pregnant after ART affirmed that their pregnancies were unintentional and came as a surprise, while health concerns and fear of transmitting HIV to a partner or a baby were the main reasons expressed by women who did not become pregnant after ART and their partners. These findings confirm our previously published quantitative results on desire for pregnancy among the cohort from which these women were selected [14] and are comparable to other studies in Uganda that report over half of the pregnancies nationally are unintended [12,14,19,20]. We also found a marked discordance between low desires for pregnancy and limited family planning use similar to what we reported quantitatively and what other authors have described in Kenya [14,21,22]. Numerous studies have reported on the barriers to family planning use in Africa [23-25] and these barriers have not changed dramatically since HIV nor since ART. Addressing some of these barriers, such as lack of access to desired methods, and poor health care providers' knowledge, experience and attitudes with these methods have enabled an increase in contraceptive use in some settings [26-29].

**Figure 3:**
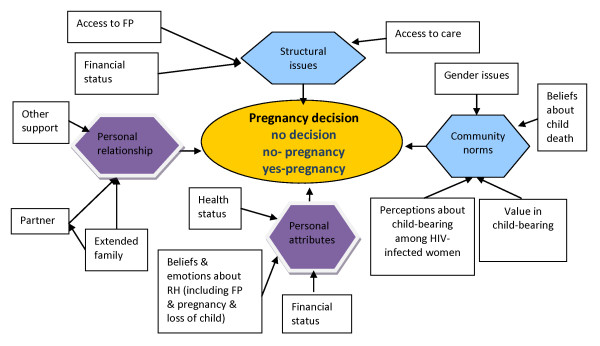
**Conceptual Framework; Influences on Pregnancy Decision-Making in HBAC Qualitative Pregnancy Study Participants; Eastern Uganda, 2006-2007**.

Our findings also highlight the numerous factors at play within individuals, couples and families that shape decisions, or in some cases, the lack of decisions around reproduction (Figure 3). Pregnancy desire among HIV-infected women has been positively associated with younger age, and knowledge of PMTCT in South Africa and Uganda [2,30] as well as with male gender, having fewer children, living in informal settlements and use of HAART in Cape Town, South Africa [31]. Our study found that when couples do make an active choice, motivations for pregnancy could be seen in two broad categories (personal/emotional and practical/structural) where numerous influences played out in different ways depending on individual context, health status, personal relationships, gender norms and beliefs as well as social/financial situations (see Figure 3; conceptual framework). For example, the role of financial status, a personal attribute, was described as a positive motivator by one man in a stable financial situation who wished to try for a male child. Whereas in a completely different context, a single woman stated that her precarious financial status pushed her to engaging in unprotected sex which resulted in an unwanted pregnancy. These findings echo what others have found in similar situations and underscore the complexity of the determinants of decisions and non-decisions [31].

As studies in South Africa and Uganda have found [2,3], our data show that HIV and ART-related health concerns including fear of MTCT of HIV, the death of a child, HIV transmission to partner and own health worries were central to some couples' decisions not to become pregnant. Other issues, were key to couples who decided not to become pregnant and were generally similar to non-HIV-infected individuals including financial constraints, reaching desired family size, not having a partner, or having a partner that one does not want to have children with [31]. Death of a child served to motivate couples to either replace lost children in some cases, or to adopt stricter family planning practices in reaction to the grieving process that was extremely painful for many. For some couples the fear of health deterioration, counseling by health providers, access to FP services and/or having reached their desired family size were sufficient factors, acting alone or in combination to motivate them to consistently use a family planning method.

Fertility desires change over time, especially in relation to health status and ART [14,15]. Reproductive health counseling for people living with HIV thus needs to emphasize the restoration of fertility after ART as an integral aspect of ongoing care[31]. In our study, several women, at the time of their ART initiation, said they could not focus on counseling about sexual activity and FP. In Uganda, ART initiation counselling is packed with information on HIV status disclosure, sero-discordance, ART drug adherence, and potential side effects as well as family planning. Individuals are often very sick at that stage and may be over-whelmed with the amount of information. This may not be the ideal time to address issues of changing fertility and family planning options. At later stages, when patients do get better and their sexual desires and sexual activity re-emerge, ART counseling focuses on drug adherence and side effects, and no longer on family planning. These are many missed opportunities to address the unmet need for family planning services. This study has thus identified a critical gap in reproductive health services for individuals in ART care. Family planning counseling and methods should be offered regularly and systematically to patients on ART throughout follow-up and more specifically in the first months following initiation of ART as they get better and are most likely to resume sexual activity.

This analysis was not without limitations. It was intended to identify influences on reproductive decisions; however as a qualitative study, we could not determine the strength of individual motivations for pregnancy. In addition, our findings are limited to individuals who were receiving HIV care, and cannot be generalized to the whole population of HIV-infected people in Uganda, the majority of whom are unaware of their HIV infection and are not on ART [32]. However, The AIDS Support Organization, where participants in this study were receiving services, was providing HIV care to over 150,000 HIV-infected clients in Uganda at the time, which represented, a significant proportion of Uganda's infected population that had been diagnosed.

## Conclusion

Our findings highlight the need for a more comprehensive approach to ART counseling that integrates maternal and child health, family planning (FP) and HIV care services in order to address the realities and clients' changing fertility desires. FP counseling would be more effective if conducted on separate, later occasions from ART initiation counseling when couples can focus on their reproductive desires in relation to newfound health. This approach will require specific elements including 1) effective integration of reproductive health especially FP and HIV care services for men and women, 2) offering dual contraceptive methods for HIV discordant couples, and 3) ensuring uninterrupted access to longer acting FP methods. Others have called for greater focus on longer-acting methods such as tubal ligation, IUD and hormonal implants after reporting similar findings[33]. This implies a greater emphasis on both research and programmatic issues such as human resources and commodities to ensure availability of comprehensive FP services and methods that cover the range of reproductive health for all HIV-infected women, men and couples. Lastly, clinicians and counselors need to be mindful that messages will only be heeded if they encompass the socio-cultural context as well as the financial and personal realities faced by persons living with HIV as they try to navigate a dramatically dynamic health situation that continuously changes the face of their social and personal worlds.

## Competing interests

The authors declare that they have no competing interests.

## Authors' contributions

RB contributed vision, design and critical comments to the manuscript. SN, KK, DK, RLK contributed in data collection design and analysis of the data. RLK contributed in overall design, analysis and manuscript writing. JH, PL, EJ contributed in study design and critical comments to the manuscript. All authors read and approved the final manuscript.

## Pre-publication history

The pre-publication history for this paper can be accessed here:

http://www.biomedcentral.com/1471-2458/11/530/prepub
